# Controllable Growth of Large–Size Crystalline MoS_2_ and Resist-Free Transfer Assisted with a Cu Thin Film

**DOI:** 10.1038/srep18596

**Published:** 2015-12-21

**Authors:** Ziyuan Lin, Yuda Zhao, Changjian Zhou, Ren Zhong, Xinsheng Wang, Yuen Hong Tsang, Yang Chai

**Affiliations:** 1 Department of Applied Physics, The Hong Kong Polytechnic University, Hung Hom, Kowloon, Hong Kong, People’s Republic of China; 2 The Hong Kong Polytechnic University Shenzhen Research Institute, Shenzhen, People’s Republic of China

## Abstract

Two-dimensional MoS_2_ is a promising material for future nanoelectronics and optoelectronics. It has remained a great challenge to grow large-size crystalline and high surface coverage monolayer MoS_2_. In this work, we investigate the controllable growth of monolayer MoS_2_ evolving from triangular flakes to continuous thin films by optimizing the concentration of gaseous MoS_2_, which has been shown a both thermodynamic and kinetic growth factor. A single-crystal monolayer MoS_2_ larger than 300 μm was successfully grown by suppressing the nuclei density and supplying sufficient source. Furthermore, we present a facile process of transferring the centimeter scale MoS_2_ assisted with a copper thin film. Our results show the absence of observable residues or wrinkles after we transfer MoS_2_ from the growth substrates onto flat substrates using this technique, which can be further extended to transfer other two-dimensional layered materials.

Two-dimensional (2D) layered materials have attracted much attention due to their unique properties^1^,^2^,^3^,^4^. The existence of a semiconductor bandgap in transitional metal dichalcogenides (TMDs), including MoS_2_, WS_2_, MoSe_2_ and WSe_2_, makes them promising for future logic devices and circuits beyond graphene. Chemical vapor deposition (CVD) has been demonstrated as a deterministic method in producing large-area and high-quality monolayer 2D layered TMD materials^5^,^6^,^7^,^8^,^9^,^10^,^11^. According to the method of placing the source materials in the furnace, the CVD growth can be divided into vapor sulfurization and vapor deposition. In the vapor sulfurization, the source materials, *e.g.,* Mo^5^, MoO_3_^6^,^7^ or (NH_4_)_2_MoS_4_ 8, are coated on a substrate, which is followed by sulfurization in a sulfur vapor environment at high temperature. The TMDs grown by sulfurization method usually have large area and good uniformity but poor crystalline quality, small domain size, and uncontrollable layer number. In contrast, the source materials, *e.g.,* MoO_3_^9^,^10^ or MoCl_5_^11^, are evaporated in the vapor deposition method. The TMDs grown by this method usually have relative large domain size but poor surface coverage on a substrate. These two kinds of CVD methods both require high growth temperature (up to ~800 °C), which cannot meet the requirements of the back-end processing of integrated circuit technology and restricts the substrate selection, *e.g.,* the plastic substrate for flexible electronics applications.

To fulfill the potential of the 2D layered TMD materials, it is important to grow large domain-size and high surface-coverage TMDs and transfer TMDs from the growth substrate to another without compromising the quality of the TMDs. The conventional PMMA-mediated transfer technique suffers from the failure of completely removing polymeric residues and the formation of the wrinkles in the TMD thin films. These drawbacks significantly degrade the performance of the TMD-based devices. Recently, Gurarslan *et al.* and Li *et al.* reported that monolayer MoS_2_ can be peeled off from the growth substrates by using polystyrene and poly(L-lactic acid) based on different surface properties between the polymers and the growth substrates^12^,^13^. These methods, compared to conventional PMMA transfer, prevents the crack of the monolayer MoS_2_ thin films during substrate etching process, but fails to avoid the use of polymer carriers, which generates polymeric residues and mechanical wrinkles. A dry transfer process for transferring graphene by mechanical delamination has been developed on the basis of different adhesion energy between the graphene on Cu foil and the target substrates^14^,^15^. However, it is easy to generate cracks on graphene during the transfer because of the adhesion between graphene and Cu foil. In addition, the use of epoxy between graphene and target substrate contaminates the surface of graphene. It is highly desirable to develop a resist-free and wrinkle-free transfer method.

In this work, we first controllably grow monolayer MoS_2_ from triangular islands to continuous thin film using vapor deposition method. After optimizing the growth parameters, we demonstrate the growth of monolayer MoS_2_ flake with large domain size (up to ~300 μm) and high surface coverage which are of great potential for variously applications. To avoid the polymeric residue and wrinkles generated in the PMMA-mediated transfer method, we present a facile transfer process using thermal release tape (TRT) assisted with a Cu thin film, preventing the MoS_2_ from direct contact to the glue and giving rise to the resist-free MoS_2_ surface. The tape and Cu thin film also provide mechanically robust supports for the monolayer MoS_2_ thin film, reducing the wrinkles generated during the transfer process. Our method allows us to transfer MoS_2_ from the growth substrate onto flat substrates without observable polymeric residues or wrinkles.

## Results and Discussion

**Figure 1:**
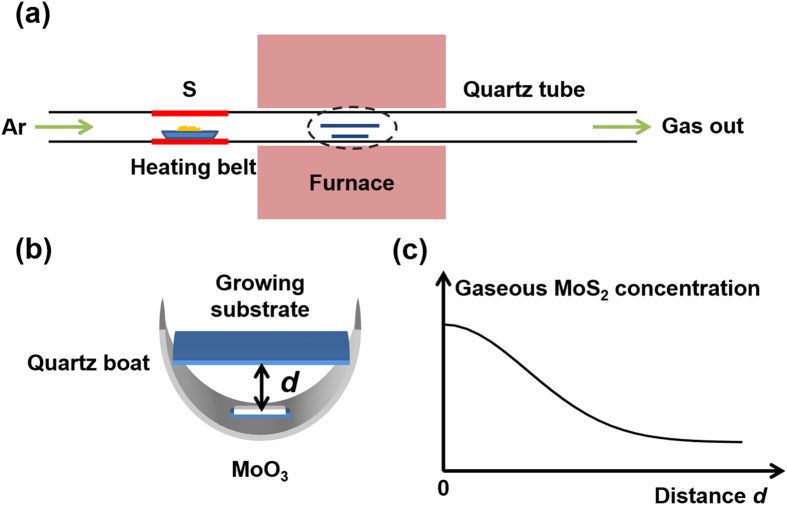
Schematic illustration of (**a**) the CVD system for growing MoS_2_ and (**b**) the quartz boat loaded in the furnace. (**c**) Schematic plot of the relationship between the gaseous MoS_2_ concentration and the distance d between MoO_3_ source and growth substrate.

We developed a CVD setup at atmospheric pressure similar to that reported in ref 16 (Fig. 1a). The temperatures of the locations for placing MoO_3_ and S precursors were controlled by the furnace and the heating belt, respectively. The sulfur gas was carried into the growth zone by Ar gas flow. Excessive amount of S were used to ensure MoO_3_ precursor react completely. In the previous works^17^,^18^, the MoO_3_ powder and the growth substrate were placed in the middle and downstream of the furnace, respectively. In our work, the growth substrate was placed on top of the quartz boat, facing downwardly towards the MoO_3_ precursor substrate, as schematically illustrated in Fig. 1b. To quantitatively control the amount of MoO_3_, we replaced commonly-used MoO_3_ powder^10^,^17^,^18^ with a MoO_3_-coated substrate as the precursor. The amount of the precursor can be adjusted by the size of the MoO_3_-coated substrates to successfully grow monolayer MoS_2_. Figure S1a shows the photograph of one typical growth substrate. Centimeter scale area of the substrate is covered with the MoS_2_ product. The optical images of the MoS_2_ at a series of points marked in Figure S1a show similar morphologies at these points, implying that the as-grown MoS_2_ is large area.

The MoS_2_ grown by vapor deposition methods are usually triangular flakes with low surface coverage, in agreement with those reported in existing literatures^17^,^19^. For the fabrication of electronic device, it is highly desirable to grow continuous and large domain size MoS_2_ thin films. The domains in the continuous MoS_2_ thin films are referred to the shape surrounded by the boundaries, in which the adjacent domains overlap at the boundary and provide abruptly optical contrast. The growth of the continuous MoS_2_ thin film is presumed to include five major steps:^11^,^18^,^20^ (1) the sublimation of the precursor (MoO_3_ and S) and the mass transport of the precursor to the region close to growth substrate, (2) the reaction of MoO_3_ and S to form gaseous MoS_2_, (3) the precipitation of supersaturated MoS_2_ vapor onto the substrate to produce solid-phase MoS_2_ nuclei, (4) the MoS_2_ growth on the substrate controlled by kinetic factors, and (5) the coalescence of the adjacent MoS_2_ domains to merge into continuous thin films.

In general, the nuclei density is closely related to the average domain size of MoS_2_. The increase of the nuclei density can improve the surface coverage but decrease the average domain size. In contrast, the suppression of nuclei density helps to grow large-size crystalline domain^21^, though the product from low-density nuclei usually has poor surface coverage. Therefore, it is quite important to optimize the nuclei density to fabricate continuous MoS_2_ thin films with large domain size as well as high surface coverage. The concentration of the gaseous MoS_2_ (*C*_*g*_) has been shown with an important thermodynamics and kinetics factor for the precipitation reaction (*C*_*g*_ → *C*_*s*_), where *C*_*s*_ is the concentration of the active species at the substrate surface^11^. In our CVD setup, the excessive S precursor outside the furnace is carried by Ar gas. The S concentration almost keeps constant in the middle of the furnace tube, and has a negligible influence on the nuclei density. On the other hand, the MoO_3_ precursor is thermally evaporated from the bottom of the quartz boat, reacts with S to form gaseous MoS_2_, and diffuses across the boundary layer towards the growth substrate. The distance between the MoO_3_ precursor substrate and the MoS_2_ growth position is defined as *d*. The relationship between the concentration of the gaseous MoS_2_ (*C*_*g*_) and the distance *d* can be described according to equation (1):^22^


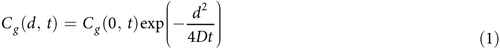


where *C*_*g*_*(d, t)* and *C*_*g*_*(0, t)* are the concentration of gaseous MoS_2_ at the given distance *d* and the source substrate, *t* is the time, and *D* is the diffusion constant. The concentration of the gaseous MoS_2_ changes dramatically as a function of *d*, as schematically plotted in Fig. 1c. This allows us to investigate the effect of the concentration of gaseous MoS_2_ on the controllable growth.

**Figure 2:**
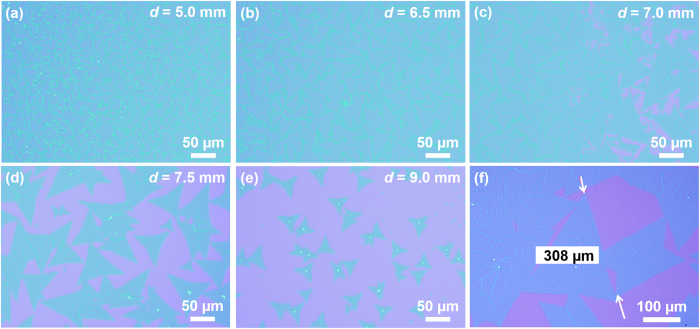
(**a–e**) Optical images of the MoS_2_ product at different places of the growth substrate. (**f**) Optical image of a crystalline MoS_2_ flake with the size of 308 μm.

Figure 2a–e show the optical images of the typical as-grown MoS_2_ with different source/substrate distances *d*. When the distance is kept relatively short (~5 mm), the concentration of the gaseous MoS_2_ *C*_*g*_ is high. Figure 2a shows that the substrate surface is fully covered by MoS_2_ thin film and the average domain size is approximately 20 μm. The domain boundaries of the thin film can be easily observed by optical microscopy because the adjacent domains overlap at the boundary and provide optical contrast. With the short distance *d*, the high *C*_*g*_ ensures that the precipitation reaction can happen and numerous sites on the surface of growth substrate can satisfy the thermodynamic conditions, resulting in high nuclei density (5.7 × 10^3^/mm^2^). When the distance increases to 6.5 mm, the MoS_2_ thin film still fully covers the growth substrate, as shown in Fig. 2b. The nuclei density rapidly decreases with lower *C*_*g*_, which allocates relatively large space for each nucleus to grow before the coalescence. As a result, the average domain size is increased to around 40 μm. Figure 2c displays the optical image of the edge of MoS_2_ thin film, where the distance *d* is around 7 mm. The overall surface coverage decreases to around 85%. In the middle part of the growth substrate, the average domain size increases to 75 μm. Triangular flakes are distributed at the edge of MoS_2_ thin films. Some overlapping flakes illustrate the initial process of the formation of the thin film. As the nuclei density is relatively low in the edge part, enough space is spare for the MoS_2_ domain to grow independently. It is worthy highlighting that the large-size crystalline MoS_2_ flake (>300 μm) has been successfully grown at this distance, Fig. 2f shows a crystalline MoS_2_ flake with the size of 308 μm. With the continuous decrease of the *C*_*g*_ at the longer distance (7.5 mm), the dominant products are triangular MoS_2_ (Fig. 2d) because the nuclei density is suppressed by the low concentration of the gaseous MoS_2_. The surface coverage is decreased to 70% and the average domain size drops to 60 μm. Low-density nuclei guarantee that most flakes can be grown independently without the overlap between the adjacent flakes until the end of the growth. When the *d* is further increased to 9 mm, the shape of the products are triangular flakes with the mean size of ~25 μm and the surface coverage is only ~30%, as shown in Fig. 2e. The low *C*_*g*_ fails to supply sufficient source, resulting in the decrease of both surface coverage and average domain size.

**Figure 3:**
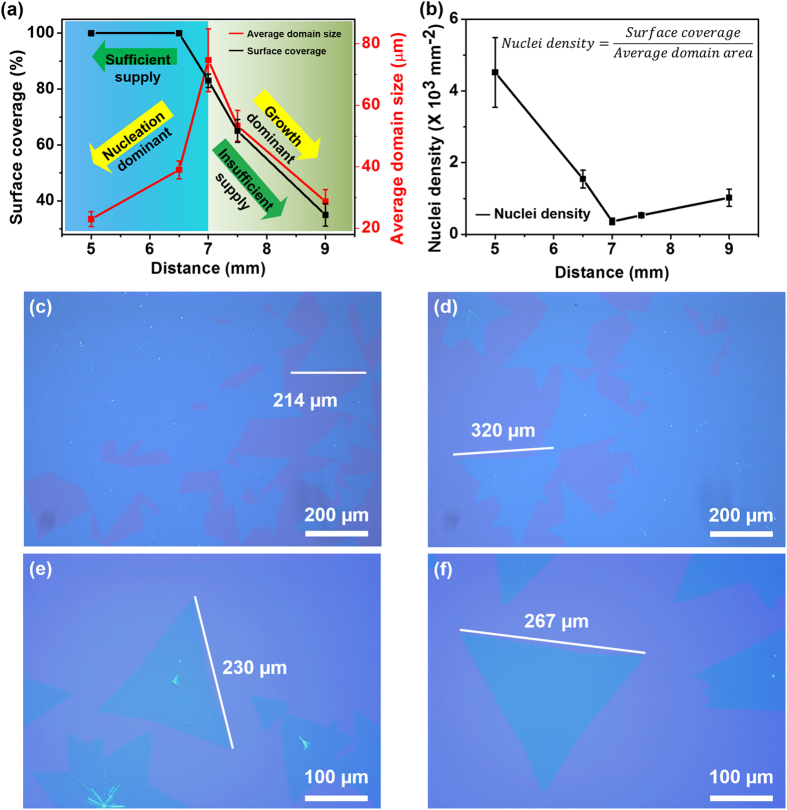
(**a**) Surface coverage and average domain size and (**b**) Nuclei density of the MoS_2_ as a function of the source/substrate distance. (**c**) to (**f**) Optical images of crystalline MoS_2_ flakes with size lager than 200 μm.

The surface coverage and the average domain size as a function of the source/growth distance are summarized in Fig. 3a. The nuclei density is calculated from 0.47 mm × 0.33 mm area and plotted in Fig. 3b. It exhibits two distinct trends in the both two plots with the distance of 7 mm as the boundary point. In the region where the growth substrate is close to the precursor substrate (less than 7 mm), the continuous MoS_2_ thin films are dominant products. The shape of the MoS_2_ domain exhibits irregular, deviating from triangle or rhomboidal. The surface coverage remains 100% and slightly decreases near the edge of MoS_2_ thin films. Meanwhile, the average domain size increases from ~23 μm to 75 μm. This is because that the high *C*_*g*_ gives rise to both high-density nuclei and sufficient source supply. Sufficient gaseous MoS_2_ supply guarantees that the high-density nuclei can keep growing until the domains merge into continuous thin films. With the distance of 7 mm, the large domain size and high surface coverage MoS_2_ thin films are grown. The average domain size of the crystalline MoS_2_ is around 75 μm in the middle part and larger than 300 μm in the edge part. The low-density nuclei help to grow large domain size without comprising the surface coverage, ascribed to the coincident match between the nuclei density and the source supply. Large-size MoS_2_ flakes over 200 μm were reproducibly grown under this condition, as shown in Fig. 3c–f.

In the range of relatively long distance (more than 7 mm), the surface coverage and average domain size both decrease with the increase of the distance. The low *C*_*g*_ results in low-density nuclei and insufficient source supply for the surface precipitation. Without sufficient gaseous MoS_2_ supply, the nuclei can grow independently during the growth procedure but fail to fully cover the surface. The insufficient MoS_2_ supply leads to smaller size of domain. Interestingly, the nuclei density is expected to decline with the increase of the distance. But a slight increase is presented in Fig. 3b. After the abrupt decrease of nuclei density, the increased spare space for each nuclei growth prevent some nuclei from being absorbed by adjacent domains and allow them to grow to observable size, which gives rise to the increase of the nuclei density.

**Figure 4:**
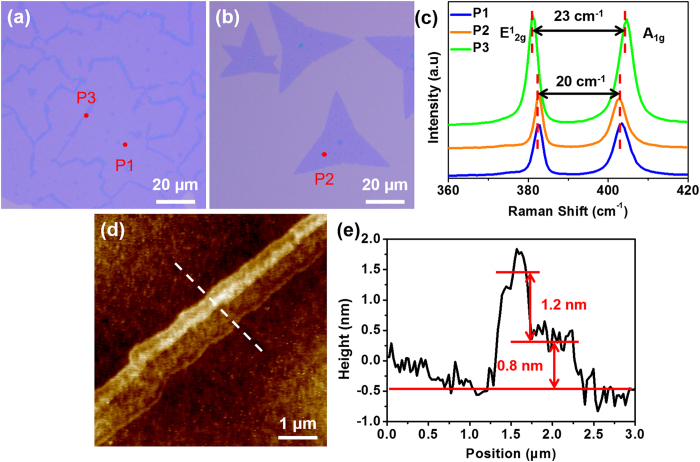
Optical images of the MoS_2_ (**a**) thin film and (**b**) flake. (**c**) Raman spectra of the MoS_2_ film, flake and boundary pointed in (**a,b**). (**d**) AFM images of MoS_2_ domain boundary. (**e**) Height profiles taken across the dash line in (**d**).

We measured the thickness of MoS_2_ thin films (Fig. 4a) and flakes (Fig. 4b) with Raman spectroscopy and atomic force microscope (AFM). Figure 4c shows the corresponding Raman spectra of the thin film and the flake. The peak difference between the in-plane mode E^1^_2g_ and out-of-plane mode A_1g_ peaks is 20 cm^−1^ at the points of P1 and P2, which represent MoS_2_ films and flakes, respectively. This indicates that the films and flakes are both monolayer^23^. The AFM images in Figure S2a and 2b show that the thickness of the MoS_2_ flake and film are ~0.8 nm and ~0.9 nm, further confirming the monolayer characteristics^4^,^9^. We also investigated the domain boundaries of the MoS_2_ thin film (P3 in Fig. 4a). The boundaries provide extra nucleation point for the MoS_2_ growth. Compared to the Raman spectra of the center of the MoS_2_ thin film, the E^1^_2g_ peak of P3 shows a red shift while the A_1g_ peak of P3 presents a blue shift. The E^1^_2g_-A_1g_ peak difference increases to 23 cm^−1^. The photoluminescence in domain boundaries region, as shown in Figure S3, also presents a much lower signal than that in the monolayer region. Figure 4d shows the AFM image of the boundary of two domains. A second layer of MoS_2_ has been grown in the boundary region and part of the MoS_2_ is 3~4 layers, which is illustrated in the corresponding height profile in Fig. 4e.

**Figure 5:**
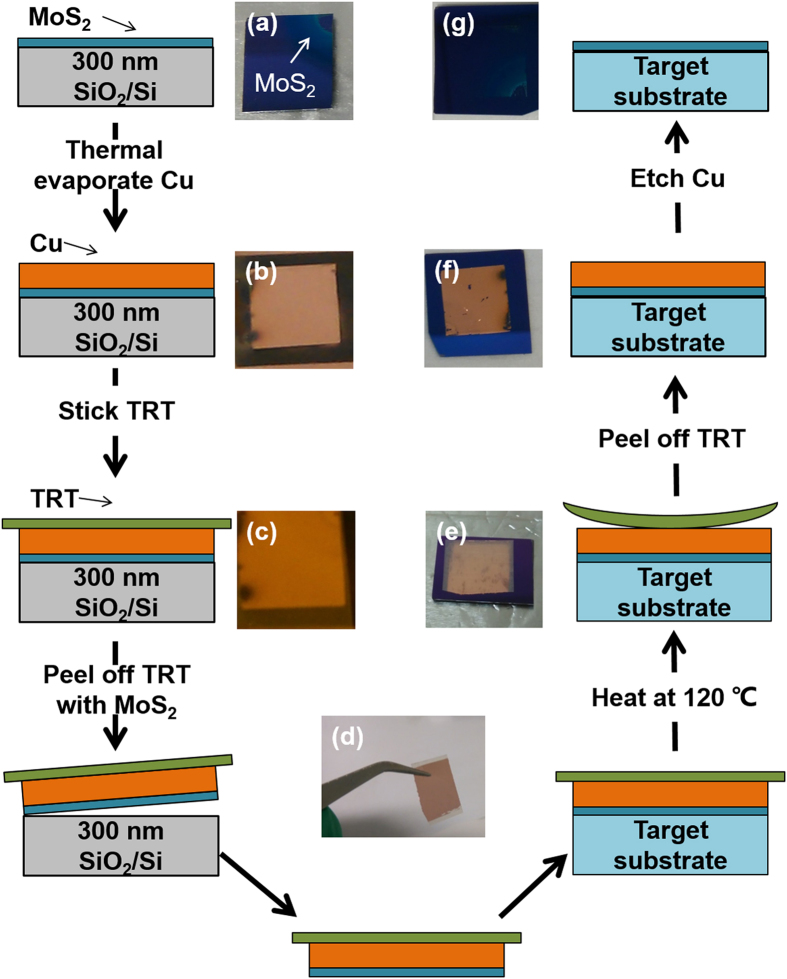
Illustration of the transfer process assisted with a Cu thin film. Photographs of the transfer processes. (**a**) as-grown MoS_2_ on SiO_2_/Si substrate; (**b**) coating the MoS_2_ with a Cu thin film; (**c**) sticking TRT; (**d**) peeling off TRT together with MoS_2_; (**e**) heating the target substrate to peel off TRT; (**f**) the MoS_2_ and Cu thin film on the target substrate; (**g**) the transferred MoS_2_ after Cu etching.

The MoS_2_ can be only grown on a few kinds of substrates (SiO_2_ or sapphire) at high temperature. To extend the applications of MoS_2_ device, it requires researchers to develop a low-temperature process to transfer MoS_2_ on various substrates, *e.g.,* ultra-high-*k* substrate or plastic substrate^24^,^25^. Figure 5 illustrates the schematics of the transfer process using thermal release tape (TRT)^26^,^27^. To prevent the direct contact between the MoS_2_ surface and the glue, MoS_2_ was first coated with a Cu thin film (~60 nm thickness) which can provide robust mechanical support and relative strong adhesion with MoS_2_ (Fig. 5b). The TRT has a strong adhesion with the Cu thin film at room temperature, which helps to completely peel MoS_2_ off from the growth substrate. The photograph in Fig. 5d clearly shows that this transfer method is applicable to centimeter-scale sample transfer. After the tape was pressed onto a target substrate, the substrate with the MoS_2_/Cu/TRT was heated from room temperature to 120 °C. The tape lost the adhesion and automatically peeled off (Fig. 5e), leaving the MoS_2_ and Cu thin film onto the target substrate (Fig. 5f). The MoS_2_ on the growth substrate (Fig. 5a, 2 cm × 2 cm) was transferred onto a 3 cm × 3 cm SiO_2_/Si substrate without notable changes (Fig. 5g). Figure S4 presents the large-area transferred film in Fig. 5g. We also transferred as-grown MoS_2_ onto SiO_2_/Si substrates by conventional PMMA mediated method as control samples^6^.

**Figure 6:**
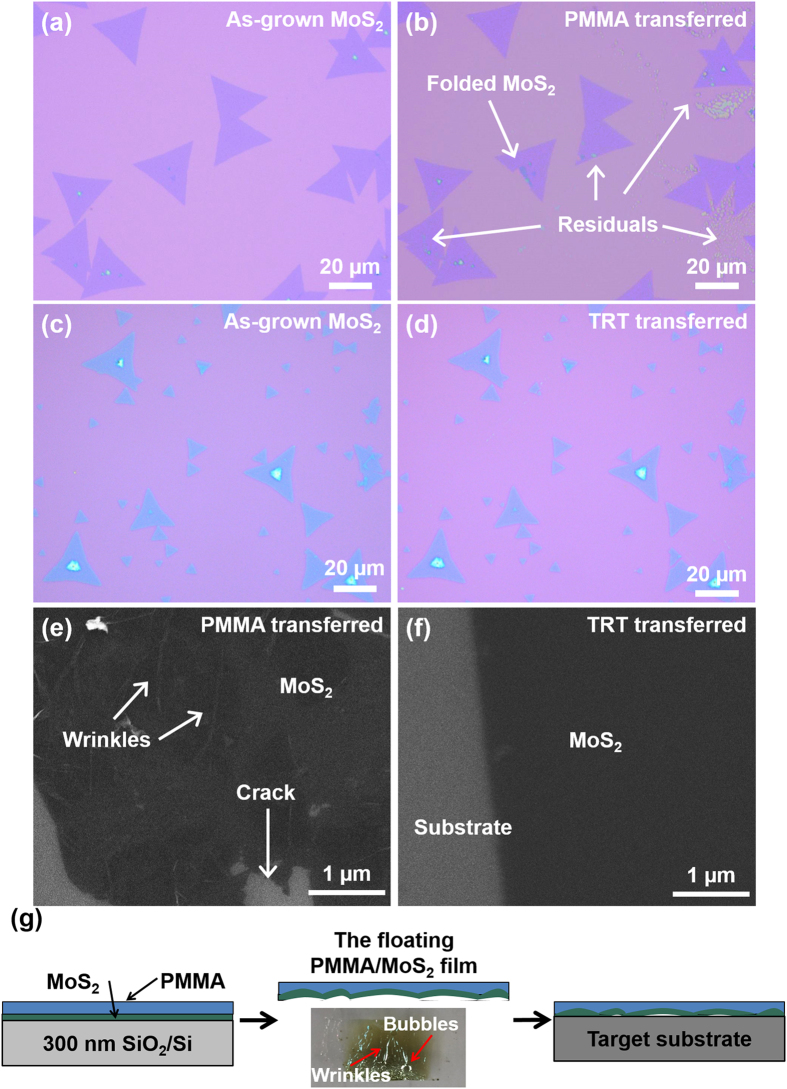
Optical images of the MoS_2_ (**a**) before and (**b**) after the PMMA-mediated transfer process. Optical images of the MoS_2_ (**c**) before and (**d**) after the TRT transfer process. SEM images of the MoS_2_ transferred by (**e**) PMMA method and (**f**) TRT method. (**g**) Illustration of wrinkle generation during PMMA transfer process and the inserted image is photograph of the PMMA/MoS_2_ film floating on the KOH solution during TRT transfer process.

Figure 6a,b show the MoS_2_ flakes before and after PMMA transfer. Compared to the as-grown MoS_2_, the PMMA-transferred MoS_2_ presents remarkable resist residues although the sample has been cleaned with acetone for long time. It has been reported that some polymeric residues are still observed on the surface of the transferred graphene when the PMMA-mediated transfer method is applied^28^,^29^,^30^,^31^,^32^. The free radicals form during PMMA cleaning process and result in the difficulty of completing removing PMMA residuals^28^. Long and heavy molecular fragments can attach to these radical sites and interact with the adjacent polymer chain. Several techniques such as chloroform treatments^29^, plasma exposure^30^ and annealing under gaseous (H_2_ and Ar) or high vacuum conditions^31^,^32^ are used to remove polymer contaminants. Though these techniques achieved some degrees of success, they are far from satisfactory for obtaining clean surface because the process flows are complicated and suffer from process specific drawbacks^33^. The harsh environment required in these techniques has uncertain influence on monolayer MoS_2_^13^, which is subject to further investigation. These polymeric residuals will degrade the performance of the electronic and optical devices based on MoS_2_. In contrast, Fig. 6c,d present the typical optical images of the as-grown and TRT transferred MoS_2_ flakes, respectively. There is no significant difference between the as-grown and the TRT transferred samples. The surfaces of the samples are clean, indicating the absence of observable resist residues. The Cu thin film introduced between MoS_2_ and TRT prevents the MoS_2_ from directly exposing to the glue. Compared to polymer, Cu thin film is relatively easier to be completely etched, which has been widely adopted for transferring graphene^34^. The EDX spectrum and XPS spectra in Figure S5 clearly show that no Cu residual exists after the etching process.

The SEM image in Fig. 6e presents the evidence of the generated wrinkles on MoS_2_ thin films after the PMMA-mediated transfer process. Figure 6f shows the SEM image of the TRT-transferred continuous MoS_2_ thin film. The flat and uniform characteristics of the transferred thin film suggest that no wrinkles were generated during our transfer process. The Young’s modulus of the Cu thin film (~100 GPa) is comparable with that of MoS_2_ (~270 GPa)^35^,^36^. It provides mechanically robust support for MoS_2_ thin films and reduces wrinkle generation during the transfer process. It also diminishes the strain induced when the TRT was peeled off during the temperature ramp process. On the contrary, PMMA has a low Young’s modulus about 22 MPa^37^, far below that of monolayer MoS_2_. Although this enables PMMA to release the growth strain, PMMA cannot provide sufficiently robust support for the MoS_2_ during the transfer process^38^. The wrinkle generation is illustrated in Fig. 6g. During the PMMA transfer process, the underlying SiO_2_ layer is etched by KOH or NaOH solution^12^. Bubbles are generated in the etching process and can be trapped by the PMMA thin film, which will induce capillary force and give rise to wrinkles or even cracks (inset image in Fig. 6g). Additionally, it is difficult to avoid the PMMA folding when we fish it out of the etching solution with target substrates. However, in our transfer technique, the Cu thin film and the TRT with robust mechanical support ensures the flatness and integrality of MoS_2_ thin film during the transfer, presented by the photography in Fig. 5d. The AFM images in Figure S6 further confirm that our TRT-transferred technique allows us to transfer large-area monolayer MoS_2_ residual-free and wrinkle-free.

**Figure 7:**
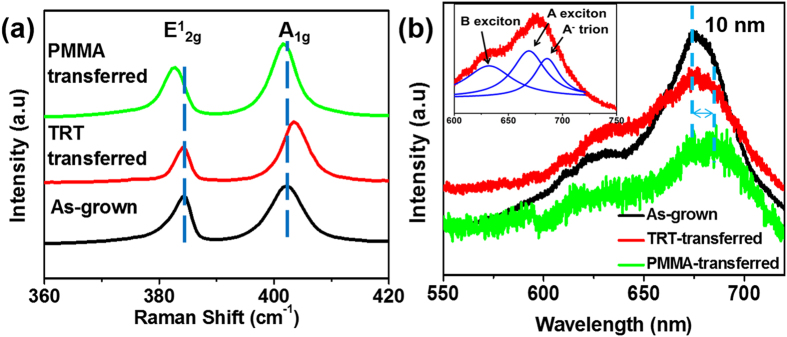
(**a**) Raman and (**b**) Photoluminescence (PL) spectra of as-grown, TRT-transferred and PMMA-transferred MoS_2_. Inserted figure is the PL spectra of TRT-transferred MoS_2_ fitted with three peaks with Lorentzian functions which are B exciton, A exciton and A- trion peaks.

Figure 7a shows the Raman spectra of the as-grown and transferred MoS_2_. The peak frequency difference between the in-plane mode E^1^_2g_ and the out-of-plane mode A_1g_ peaks remains around 19~20 cm^−1^ after the transfer by PMMA and TRT methods, indicating that the layer number remains monolayer^23^. The peak position of the in-plane mode E^1^_2g_ in Raman spectra is sensitive to the strain and can be used as an indicator of the strain^39^. The peak position remains unchanged before and after the TRT transfer. However this peak redly shift about 2 cm^−1^ after the transfer by PMMA, which results from the local strain induced by the wrinkle generated during the transfer^40^. This is also in agreement with our morphology characterization. The peak position of the out-of-plane mode A_1g_ is less affected by the strain than the in-plane E^1^_2g_ mode^39^. The slight down-shift of the A_1g_ mode of the MoS_2_ by PMMA-mediated transfer method also indicates the presence of the strain after the transfer^40^. However, the A_1g_ mode upshifts after the TRT transfer shows a different trend. The stiffening A_1g_ mode is associated with the p-doping in the MoS_2_^41^, coming from the charged impurities, which is unavoidably introduced at the interface between the MoS_2_ and the target substrates during the transfer process^42^. This impurities also exist in PMMA-mediated transferred MoS_2_, but the doping yields to the effect of the strain and fails to stiffen A_1g_ mode^40^.

Figure 7b shows the photoluminescence (PL) spectra of the MoS_2_ samples. After PMMA-mediated transfer, two striking PL changes are observed: first, the emission is shifted by 10 nm to lower energy; second, the intensity is rapidly decreased. As PMMA cannot provide mechanical robust support for the MoS_2_, it is unavoidable to introduce local strain into MoS_2_ samples during the PMMA-mediated transfer process. It has been reported that the uniaxial strain on monolayer MoS_2_ gives rise to the red shift of PL peak and the reduced peak intensity due to the change of band structure and the reduction in the band gap energy^40^,^43^,^44^, which is also observed in our work. And the change of band structure is attributed to the weak PL signal of PMMA-transferred MoS_2_^43^. In contrast, the PL peak positions of the TRT-transferred MoS_2_ are comparable to that of the as-grown MoS_2_ in terms of peak position. However, the peak intensity shows a slightly decrease and the full width at half maximum (FWHM) increases, which is induced by charge doping as we observe from the Raman results. The direct-gap transition peak (A peak) can be fitted with two peaks: A exciton peak and A^−^ trion peak^45^, shown in the inserted figure in Fig. 7b. The A^−^ trion peak is sensitive to the electron density and the intensity will decrease by p-doping^46^. The A^−^ trion intensity of TRT-transferred MoS_2_ decreases due to the impurities at the interface, making the principal peak slightly lower than that of as-grown MoS_2_.

## Conclusions

We fabricated MoS_2_ triangular flakes and continuous thin films by changing the distance between source and growth substrates. The effects of the nuclei density on the domain size and surface coverage were systematically investigated. By optimizing the growth conditions, we have successfully grown single-crystalline MoS_2_ flake with the size larger than 300 μm. We also developed a transfer process assisted with a Cu thin film, which allows us to transfer MoS_2_ onto flat substrates. The optical and SEM images confirmed the absence of observable residues and wrinkles on the transferred MoS_2_. The properties were measured by Raman and PL suggesting the transferred MoS_2_ maintained qualities as the as-grown one. Although this work focused on the transfer of MoS_2_, this process can be extended to the transfer of other 2D layered materials.

## Methods

### MoO_3_-coated substrate preparation

MoO_3_ was prepared by conventional hydrothermal synthesis^47^ and fully dissolved in ethanol. The mixture was then dropped onto a Si substrate with 300-nm-thick SiO_2_ and the substrate was heated to approximately 110 °C on a hot plate to evaporate ethanol. The procedure was repeated until the remaining MoO_3_ completely covered the substrate.

### CVD method

MoS_2_ was grown on a Si substrate with 300-nm-thick SiO_2_ by CVD method. The growth substrate faced downwardly above a quartz boat. Below the growth substrate, a 5 mm × 5 mm MoO_3_-coated substrate (about 10-30 mg MoO_3_) was placed as the Mo precursor. The quartz boat was then loaded into a 1-inch-diameter quartz tube and centered in the furnace. A porcelain boat with sulfur was located in the upstream region outside the furnace, approximately 25 cm away from the middle. Before the MoS_2_ growth, the system was flushed with 200 sccm of Ar gas for 10 min. Afterwards, the MoO_3_ was heated from room temperature to ~800 °C at a rate of 20 °C/min and then maintained at 800 °C for 10 min. Meanwhile, the sulfur was sublimated at 160 °C and carried by Ar gas flow to the growth zone. The Ar gas flow was initially 150 sccm, and when the growth region reaches 700 °C, it was reduced to 60 sccm to prevent excessive sulfur from suppressing the evaporation of the Mo source. The growth ended with natural cooling of the system.

### Transfer by thermal release tape

The substrate with as-grown MoS_2_ sample was first coated with a ~60-nm-thick Cu thin film by thermal evaporation. A thermal release tape was then pressed onto the SiO_2_/Si substrate and was gently peeled off. As a result, the Cu/MoS_2_ stack was separated from the SiO_2_/Si substrate and attached to the tape. Second, the tape carrying Cu/MoS_2_ was pressed onto a targeted substrate. The whole stack was heated to approximately 120 °C. The adhesiveness between thermal release tape and Cu was weakened. Therefore the tape can be easily peeled off, leaving the Cu/MoS_2_ on the target substrate. Finally, the Cu thin film was etched using the mix of 15% ammonium persulphate and deionization water, followed by a modified RCA clean process^34^.

## Additional Information

**How to cite this article**: Lin, Z. *et al.* Controllable Growth of Large–Size Crystalline MoS_2_ and Resist-Free Transfer Assisted with a Cu Thin Film. *Sci. Rep.* **5**, 18596; doi: 10.1038/srep18596 (2015).

## Supplementary Material

Supplementary Information
