# Supplementary material for: Controllable Growth of Large–Size Crystalline MoS2 and Resist-Free Transfer Assisted with a Cu Thin Film
Source: Sci Rep. 2015 Dec 21;5:18596. doi: 10.1038/srep18596 (PMC4685646; doi:10.1038/srep18596)
Supplement: Supplementary Information [file srep18596-s1.pdf]

Supporting Information

Controllable Growth of Large–Size Crystalline MoS2 and Resist-Free Transfer Assisted with a Cu Thin Film

Ziyuan Lin,[1] *Yuda Zhao,*[1] *Changjian Zhou,*[1] *Ren Zhong,*[1] *Xinsheng Wang,*[1] *Yuen Hong Tsang,*[1] *and Yang Chai*[1,2*]

[1] Department of Applied Physics, The Hong Kong Polytechnic University, Hung Hom, Kowloon, Hong Kong, People's Republic of China.

[*]Corresponding author: ychai@polyu.edu.hk

[2] The Hong Kong Polytechnic University Shenzhen Research Institute, Shenzhen, People's Republic of China

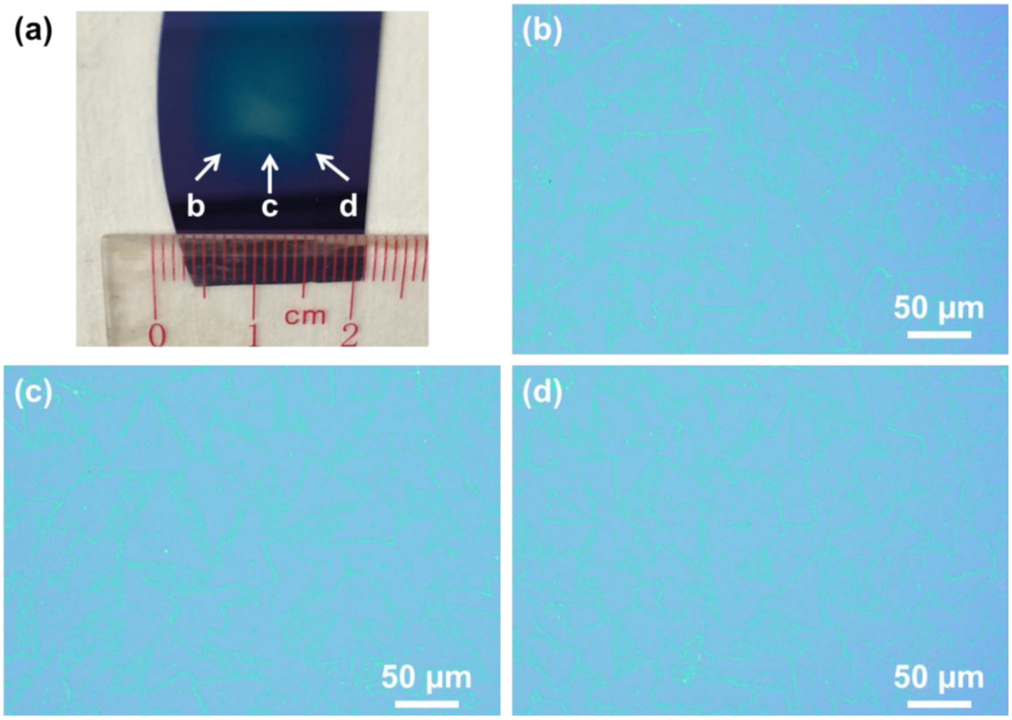

Figure S1. (a) Photographs of one typical growth substrate. (b) to (d) Optical images of the MoS_2 product at different places marked in (a).

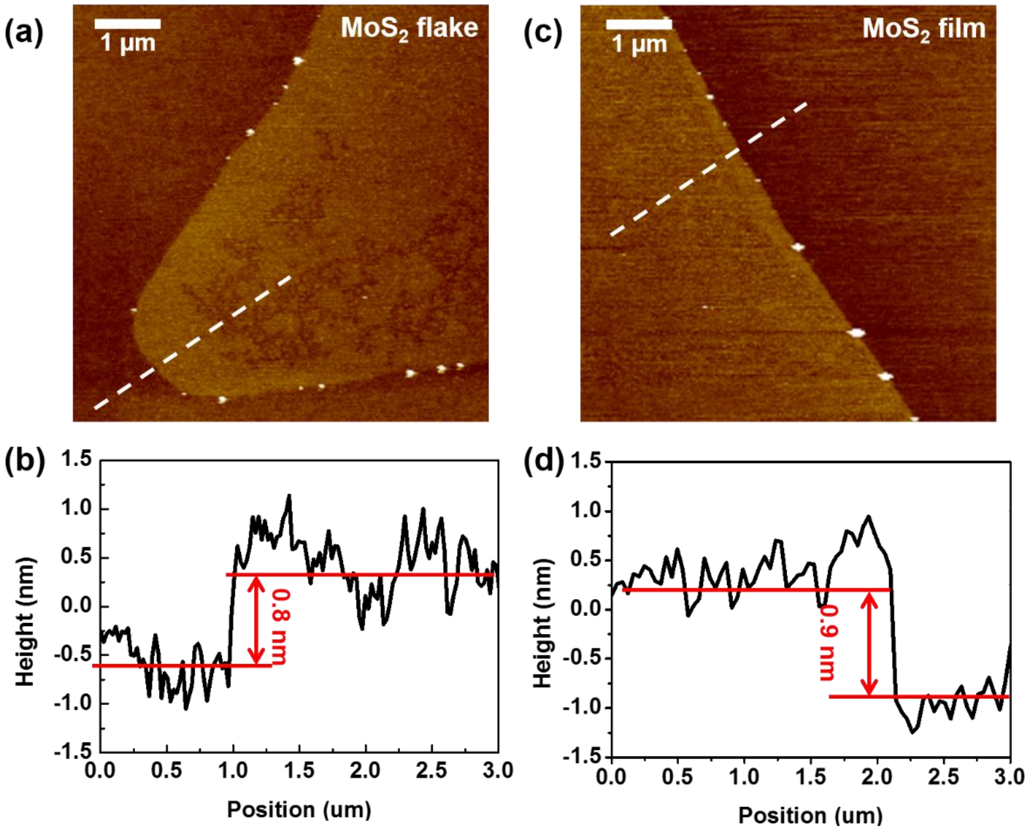

Figure S2. (a and c) AFM images of MoS_2 triangular flake and thin film. (b and d) Height profiles taken across the dash line in (a and c).

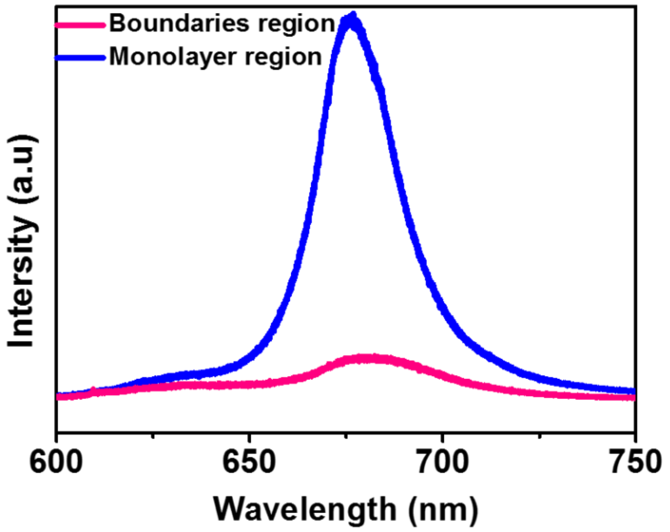

Figure S3. Photoluminescence (PL) spectra of the crystalline MoS_2 in the monolayer region and the domain boundaries region.

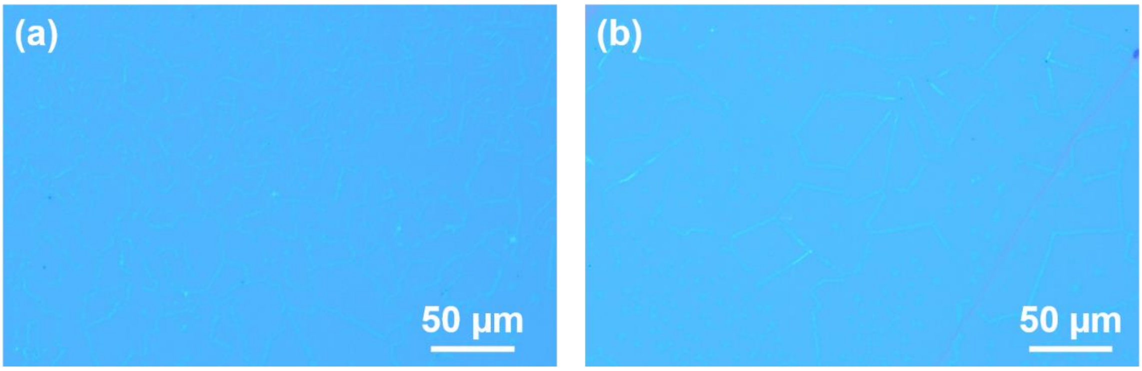

Figure S4. Optical images of large area MoS_2 film transferred by thermal release tape method taken from one sample.

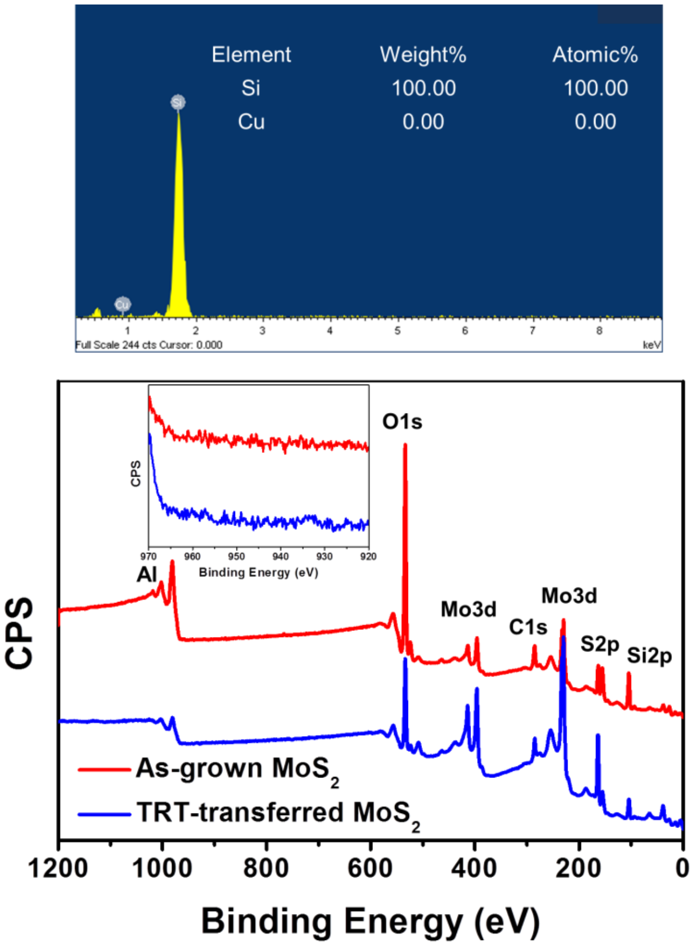

Figure S5. (a) EDX spectra of the MoS_2 transferred by thermal release tape method with the element quantification results. (b) XPS spectra of the MoS_2 before and after thermal release tape transfer method. Inserted are the XPS spectra of the Cu 2p region.

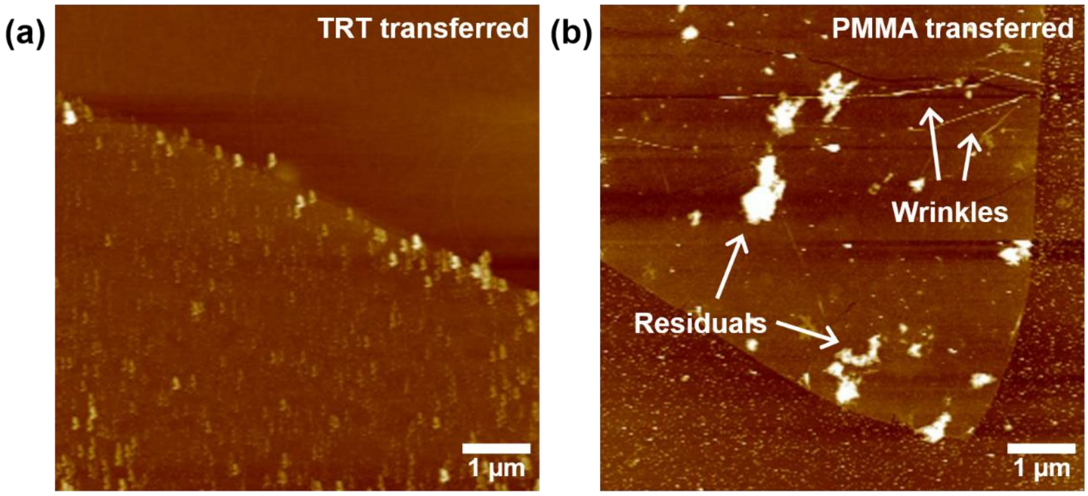

Figure S6. (a and b) AFM images of MoS_2 transferred by thermal release tape method and PMMA method.
